# Hypertension and associated factors in older adults in South Africa

**DOI:** 10.5830/CVJA-2013-002

**Published:** 2013-04

**Authors:** Karl Peltzer, Nancy Phaswana-Mafuya

**Affiliations:** HIV/AIDS/SIT and TB (HAST), Human Sciences Research Council, Pretoria, South Africa; Department of Psychology, University of Limpopo, Turfloop, South Africa and ASEAN Institute for Health Development, Mahidol University, Salaya, Thailand; HIV/AIDS/SIT and TB (HAST), Human Sciences Research Council, Pretoria, South Africa; Office of the Vice Chancellor, Nelson Mandela Metropolitan University, Port Elizabeth, South Africa

**Keywords:** hypertension, risk factors, older adults, South Africa

## Abstract

**Background:**

Older adults are disproportionately affected by hypertension, which is an established risk factor for cardiovascular disease. Little attention has been focused on hypertension and associated factors among older adults in Africa. Therefore, this study aimed to investigate the prevalence and associated factors of hypertension in a national sample of older South Africans who participated in the Study of Global Ageing and Adults’ Health (SAGE) in 2008.

**Methods:**

In 2008 we conducted a national, population-based, cross-sectional study of a sample of 3 840 subjects aged 50 years or older in South Africa. The questionnaire included socio-demographic characteristics, health variables, and anthropometric and blood pressure measurements.

**Results:**

The prevalence of hypertension in the sample population was 77.3% (male 74.4%, female 79.6%). The rates of awareness, treatment and control among the hypertensive participants were 38.1, 32.7 and 17.1%, respectively. The results of multivariate logistic regression analysis revealed that the prevalence of hypertension was associated with being in the Coloured population group, having had a stroke, being overweight or obese and having had five or more out-patients care visits in the past 12 months. Hypertension was inversely associated with current alcohol use.

**Conclusion:**

This study revealed high rates of hypertension among older adults (50 years and more) in South Africa, which puts them at risk for cardiovascular disease. The percentages of hypertensive subjects who were aware, treated and controlled were very low. These data underscore the urgent need to strengthen the public health education and blood pressure-monitoring systems to better manage hypertension among older adults in South Africa.

## Abstract

High blood pressure in South Africa is estimated to have caused 46 888 deaths and 390 860 disability-adjusted life years in 2000.^1^ Hypertension alone is the leading reason for attending primary care and is the most common diagnosis (13.1%) in South Africa.^2^ In population-based surveys, high rates of hypertension were found among older adults in South Africa, 44.0–52.0% among men and 51.6–60.4% among women in 1998.^3^

In other countries, in Dakar, Senegal, 65.4% of subjects had hypertension,^4^ in urban Zimbabwe 72%.^5^ In Malawi, Rwanda and Tanzania, hypertension was found in 41.0% of men and 36.6% of women.^6^ In Costa Rica, 65% were hypertensive,^7^ and 42.4% of women in Accra, Ghana had hypertension.^8^ In Brazil, self-reported hypertension was 55%,^9^ in rural China it was 57–64.9%,^10^ and in another study in China it was 24.2–43.8%.^11^ In Turkey 71.2–82.2% of subjects had hypertension,^12^ and in Taiwan 31.1–38.0%.^13^

Various factors have been found to be associated with hypertension, including socio-demographics (older age, female gender, lower education level, lower household income),^13^-^16^ geolocality (urban residence),^17^ other risk factors or behaviour, including stroke,^18^ diabetes,^19^,^20^ higher body mass index (BMI),^5^,^10^,^13^,^14^ physical inactivity,^16^,^22^,^23^ insufficient fruit and vegetable intake,^13^,^23^-^25^ smoking and drinking,^10^,^16^,^22^ greater limitations on activities of daily living (ADLs) and instrumental activities of daily living (IADLs),^26^ higher frequency of doctor visits,^4^ and less social cohesion.^27^

In general, it is estimated that in South Africa only 26% of men and 51% of women are aware of their hypertension.^1^ Among older adults in Senegal, half of those suffering from high blood pressure were aware of their problem, and among the latter, 70% said they were on treatment. However, of these, only 17% had controlled arterial blood pressure.^4^ Among rural older adults in China, the rates of awareness, treatment and control were very low (overall 35.2, 28.7 and 1.0%, respectively).^10^

Few studies exist investigating hypertension among older adults in low- and middle-income countries. Yet, research studies ‘demonstrate a clear evolution in the prevalence, awareness, treatment and control of hypertension during the ageing process’.^4^ Therefore, this study aimed to investigate the prevalence and associated factors of hypertension in a national sample of older South Africans who participated in the Study of Global Ageing and Adults’ Health (SAGE) in 2008.

## Methods

We conducted a national, population-based, cross-sectional study with a sample of 3 840 subjects aged 50 years or older in South Africa in 2008. The SAGE sample design entailed a two-stage probability sample that yielded national and sub-national estimates to an acceptable precision at provincial level, by locality type (urban and rural) and population group (including black, Coloured, Indian or Asian, and white). The overall response rate among those aged 50 years or older was 60%.

The Global Study on Ageing (SAGE) survey was carried out in South Africa in partnership with the World Health Organisation (WHO), the National Department of Health, and the Human Sciences Research Council (HSRC). The study was approved by the Human Sciences Research Council Research Ethics Committee and the national Department of Health.

Blood pressure (systolic and diastolic) was measured three times on the right arm/wrist of the seated respondent using an automated recording device (OMRON R6 Wrist Blood Pressure Monitor, HEM-6000-E, Omron Healthcare Europe, BV, Hoofddorp and The Netherlands).

Out of three measurements, the average of the last two readings was used. In accordance with the Seventh Report of the Joint National Committee on Prevention, Detection, Evaluation and Treatment of High Blood Pressure, individuals with systolic blood pressure ≥ 140 mmHg and/or diastolic blood pressure ≥ 90 mmHg and/or who reported the current use of antihypertensive medication were considered to be suffering from high blood pressure.28

Awareness was defined as history of hypertension based on diagnosis by a healthcare provider. Treatment was defined as taking any medication or other treatment for hypertension in the last two weeks prior to the survey, and control was defined as blood pressure < 140 and < 90 mmHg at the time of the survey.

Lifetime tobacco users were asked ‘Do you currently use (smoke, sniff or chew) any tobacco products such as cigarettes, cigars, pipes, chewing tobacco or snuff?’ The response options were ‘Yes, daily’, ‘Yes, but not daily’ and ‘No, not at all’. Daily tobacco use was coded = 1, and not daily and not at all = 0.^29^

Lifetime alcohol users were asked about current (past month) alcohol use. Past month alcohol use was coded = 1 and no past month alcohol use = 0.

Height and weight were measured. Body mass index (BMI) was used as an indicator of obesity (≥ 30 kg/m^2^), calculated as weight in kg divided by height in metres squared. Overweight and/or obesity were defined as BMI ≥ 25 kg/m^2^ and underweight as < 18.5 kg/m^2^.

Social cohesion was measured with nine items, starting with the introduction ‘How often in the last 12 months have you… e.g. attended any group, club, society, union or organisational meeting?’ All nine items were summed to get a social cohesion index. Response options ranged from never = 1 to daily = 5. Cronbach alpha for the social cohesion index in this sample was 0.73.

Physical activity was measured using the General Physical Activity Questionnaire (GPAQ). The instrument gathers information on physical activity in three domains (activity at work, travel to and from places, and recreational activities), as well as time spent sitting. The questionnaire also assesses vigorous and moderate activities performed at work and for recreational activities. Information on the number of days a week spent on different activities, and time spent in a typical day for each activity was also recorded.^30^ Cronbach alpha for the GPAQ in this sample was 0.77.

For physical activity, in addition to the total minutes of activity, the activity volume was also computed by weighting each type of activity by its energy requirement in metabolic equivalents (METs). One MET was defined as the energy cost of sitting quietly, and was equivalent to a caloric consumption of 1 kcal/kg/h. A MET-minute showed the total activity volume on a weekly basis, and was calculated by multiplying the time spent on each activity during a week by the MET-values of each level of activity. MET-values for different levels of activities were set as 4 MET for moderate intensity physical activity, 8 MET for vigorous physical activity, and 4 MET for transport-related walking or cycling.

The total physical activity for GPAQ2 was calculated as the sum of the total moderate, vigorous, and transport-related activities per week. The number of days and total physical activity MET-minutes per week were used to classify respondents into three categories of low, moderate and high level of physical activities. Less than 600 MET-minutes per week was classified as low physical activity.^30^

Fruit and vegetable consumption was assessed with the questions ‘How many servings of fruit do you eat on a typical day?’ and ‘How many servings of vegetables do you eat on a typical day?’ Insufficient fruit and vegetable consumption was defined as less than five servings of fruits and/or vegetables a day.

Overall self-rated health status was based on respondents’ assessment of their current health status on a five-point scale in response to the question: ‘In general, how would you rate your health today?’ Response categories were: very good, good, moderate, bad and very bad. Very good and good were grouped together and coded = 1, moderate = 2 and bad and very bad were grouped together and coded = 3.

Activity limitation (difficulty an individual may have in executing task or actions) was assessed with one item ‘Overall in the last 30 days, how much difficulty did you have with work or household activities?’ Response options ranged from 1 = none to 5 = extreme/cannot do. None were coded = 1, mild = 2, moderate = 3 and severe and extreme were grouped together and coded = 4.

Finally, participants were asked about a list of chronic and other conditions they had been diagnosed with, including diabetes, hypertension, stroke, angina and arthritis. Of participants who responded to having been diagnosed with hypertension, the question was asked, ‘Have you been taking any medication or other treatment for it during the last two weeks?’ Other treatment might include a weight-loss programme or change in eating habits.

Attendance at out-patient care was assessed with the question, ‘Over the last 12 months, did you receive ant healthcare not including an overnight stay in hospital or long-term care facility?’ Of those who indicated ‘yes’ they had to report the number of times they had received healthcare or consultation in the last 12 months. Frequency of attendance at out-patient care was grouped into none = 0, one to four = 2, five or more = 3.

To estimate economic or wealth status, a random-effects probit model was used to identify indicator-specific thresholds that represent the point on the wealth scale above which a household is more likely to own a particular asset than not. This enabled an estimation of an asset ladder. These estimates of thresholds, combined with actual assets observed to be owned for any given household, were used to produce an estimate of household-level wealth status. This was used to create wealth quintiles.^31^ Lowest and second-lowest wealth quintiles were grouped together as low = 1, the middle wealth quintile was medium = 2 and the fourth and highest wealth quintiles were grouped together as high = 3.

## Statistical analysis

The data were entered using CSPro and analysed using STATA Version 10. The data were weighted using post-stratified individual probability weights based on the selection probability at each stage of selection. Individual weights were post-stratified by province, gender and age groups according to the 2009 medium mid-year population estimates from Statistics South Africa, available at: http://www.statssa.gov.za/publications/P0302/P03022009.pdf.

Computed estimates and odds ratios were reported with 95% confidence intervals and a two-tailed p-value of 0.05 was used as the cut-off point for statistical significance. Associations between key outcomes of hypertension, and socio-demographic and health variables were evaluated calculating odds ratios (OR).

Multivariate logistic regression was used for evaluation of the impact of explanatory variables for key outcome of hypertension (binary dependent variable). All variables statistically significant at *p* < 0.05 in bivariate analyses were included in the multivariable models. In the analysis, weighted percentages were reported.

## Results

The total sample included 3 840 South African subjects 50 years or older, 44.1% were men and 55.9% were women. The most prevalent population group was black Africans (74%), and almost half (49.9%) were between 50 and 59 years old. The educational level of most participants (71.6%) was lower than secondary school education and almost two-thirds (64.9%) lived in an urban area.

A large group (72.4%) of older adults were overweight or obese, 20.4% were daily tobacco users, 4.0% had had a stroke, and 9.2% had diabetes. More than half (52.2%) engaged in low physical activity, two-thirds (67.7%) ate insufficient fruit and vegetables, and a small proportion (13.7%) were current alcohol users. A sizeable proportion (17.5%) rated their health status as bad or very bad, 10.7% reported severe or extreme activity limitation and 28.1% were out-patients five times or more. The prevalence rates of hypertension were 77.3% (male 74.4%, female 79.6%) (Table 1).

**Table 1:** Sample Characteristics And Prevalence Rate Of Hypertension Among Older South Africans

		*Prevalence rate of hypertension*		
	*Total sample*	*Male (n = 1638)*	*Female (n = 2202)*	*Total*
All	3840	1159 (74.4)	1683 (79.6)	2842 (77.3)
Age (years)				
50–59	1695 (49.9)	520 (71.3)	682 (77.9)	1202 (74.9)
60–69	1233 (30.6)	391 (79.4)	563 (81.6)	954 (80.6)
70 and over	912 (19.5)	248 (75.1)	438 (80.5)	686 (78.4)
Population group				
African black	2053 (74.0)	575 (73.1)	975 (80.0)	1550 (77.3)
White	269 (9.3)	92 (75.6)	101 (83.0)	193 (79.6)
Coloured	655 (12.8)	195 (88.1)	350 (83.1)	545 (85.0)
Indian or Asian	307 (3.8)	97 (74.1)	117 (78.5)	214 (76.8)
Marital status				
Single	512 (14.3)	97 (71.5)	292 (83.1)	389 (80.1)
Married	2007 (55.9)	870 (74.2)	571 (74.2)	1441 (74.2)
Separated/divorced	230 (5.9)	60 (66.0)	116 (82.3)	176 (77.7)
Widow	1020 (23.9)	120 (84.8)	667 (82.6)	787 (82.9)
Educational level				
No schooling	854 (25.2)	231 (78.3)	422 (81.2)	653 (80.2)
Less than primary	803 (24.0)	227 (69.4)	404 (85.6)	631 (78.9)
Primary	779 (22.4)	232 (79.2)	364 (79.3)	596 (79.2)
Secondary	923 (28.3)	261 (74.9)	337 (76.5)	598 (75.8)
Wealth				
Low	1482 (40.6)	429 (71.1)	646 (78.7)	1075 (75.4)
Medium	731 (18.2)	197 (74.7)	377 (79.9)	574 (78.3)
High	1608 (41.2)	525 (77.1)	651 (80.2)	1176 (78.6)
Geolocality				
Rural	1276 (35.1)	392 (75.9)	524 (78.7)	916 (77.5)
Urban	2561 (64.9)	766 (73.7)	1157 (80.1)	1923 (77.2)
Other conditions				
Stroke	139 (4.0)	48 (88.2)	71 (91.5)	119 (90.0)
Angina	219 (5.2)	62 (76.2)	114 (76.2)	176 (76.2)
Diabetes	360 (9.2)	107 (88.4)	202 (90.2)	309 (89.6)
Overweight (BMI ≥ 25 kg/m^2^)	2505 (72.4)	745 (77.3)	1253 (82.8)	1998 (80.5)
Underweight (BMI < 18.5 kg/m^2^)	184 (4.3)	51 (64.4)	63 (66.8)	114 (65.5)
Arthritis	851 (24.7)	198 (81.8)	472 (83.0)	670 (82.6)
Daily tobacco use	810 (20.4)	295 (74.6)	315 (76.3)	610 (75.4)
Alcohol use (past month)	557 (13.7)	248 (66.6)	151 (77.5)	399 (70.3)
Physical activity				
Low	2100 (52.2)	567 (73.2)	940 (79.0)	1507 (76.6)
Moderate	692 (16.6)	233 (79.6)	302 (76.9)	535 (78.1)
High	1044 (31.2)	357 (73.4)	440 (82.1)	797 (78.1)
Insufficient fruit and vegetables	2817 (67.7)	834 (77.3)	1245 (77.0)	2079 (77.1)
Subjective health status				
Very/good	1469 (37.9)	486 (71.3)	565 (75.4)	1051 (73.4)
Moderate	1681 (44.9)	495 (78.2)	834 (80.9)	1329 (79.8)
Bad/very bad	617 (17.5)	177 (73.9)	281 (84.4)	458 (79.9)
Activity limitation				
None	1465 (38.5)	487 (73.6)	566 (74.8)	1053 (74.2)
Mild	625 (16.7)	191 (71.6)	297 (80.5)	488 (76.5)
Moderate	1275 (34.2)	369 (75.9)	628 (81.0)	997 (78.9)
Severe/extreme	370 (10.7)	103 (78.5)	178 (89.6)	281 (85.8)
Social cohesion index (range 9–72); mean (SD)	22.1 (6.5)	21.9 (6.2)	21.4 (6.1)	21.6 (6.1)
Outpatient visits in past 12 months				
0	1176 (38.2)	372 (72.2)	464 (74.5)	836 (73.3)
1–4	908 (33.7)	271 (76.2)	414 (79.2)	685 (77.9)
5 or more	941 (28.1)	282 (80.4)	513 (86.4)	795 (84.2)

The results of the multivariate logistic regression analysis revealed that the prevalence of hypertension was associated with being in the Coloured population group, having had a stroke, being overweight and having had five or more out-patients care visits in the past 12 months. Prevalence of hypertension was inversely associated with current alcohol use (Table 2).

**Table 2:** Multiple Logistic Regression Of Hypertension Prevalence In Older South Africans

	*UOR (95% CI)*	*AOR (95% CI)*
Gender	
Female	1.00	1.00
Male	0.75 (0.57–0.98)*	0.85 (0.80–1.63)
Age (years)	
50–59	1.00	1.00
60–69	1.40 (1.12–1.75)**	1.30 (0.94–1.79)
70 and over	1.22 (0.87–1.70)	1.19 (0.80–1.78)
Population group	
African black	1.00	1.00
White	1.14 (0.64–2.05)	1.23 (0.66–2.30)
Coloured	1.66 (1.07–2.59)*	1.89 (1.04–3.44)*
Indian or Asian	0.97 (0.59–1.60)	0.82 (0.47–1.42)
Marital status	
Single	1.00	
Married	0.71 (0.43–1.18)	
Separated/divorced	0.86 (0.43–1.72)	
Widow	1.20 (0.66–2.19)	
Educational level	
No schooling	1.00	
Less than primary	1.15 (0.74–1.81)	
Primary	1.18 (0.86–1.60)	
Secondary	0.97 (0.71–1.31)	
Wealth	
Low	1.00	
Medium	1.18 (0.78–1.77)	
High	1.20 (0.80–1.80)	
Geolocality	
Rural	1.00	
Urban	0.98 (0.59–1.65)	
Other conditions	
Stroke	2.67 (1.10–6.47)*	4.48 (1.48–13.59)**
Angina	0.92 (0.56–1.51)	
Diabetes	2.65 (1.86–3.78)***	1.30 (0.86–1.98)
Overweight(BMI ≥ 25 kg/m^2^)	1.67 (1.41–1.98)***	1.52 (1.15–2.01)**
Underweight (BMI < 18.5 kg/m^2^)	0.53 (0.30–0.97)*	0.77 (0.36–1.64)
Arthritis	1.49 (1.06–2.11)*	1.13 (0.75–1.69)
Daily tobacco use	0.85 (0.56–1.30)	
Alcohol use (past month)	0.64 (0.47–0.85)**	0.64 (0.49–0.84)**
Physical activity	
Low	1.00	
Moderate	1.01 (0.70–1.45)	
High	0.92 (0.66–1.27)	
Insufficient fruit and vegetables	0.96 (0.65–1.43)	
Subjective health status	
Very/good	1.00	1.00
Moderate	1.43 (1.12–1.83)**	1.41 (0.95–2.10)
Bad/very bad	1.44 (0.77–2.68)	1.27 (0.62–2.58)
Activity limitation	
None	1.00	1.00
Mild	1.13 (0.84–1.52)	0.92 (0.59–1.45)
Moderate	1.30 (0.90–1.88)	0.92 (0.58–1.48)
Severe/extreme	2.10 (1.12–3.93)*	1.60 (0.70–3.64)
Social cohesion index (range 9–72); mean (SD)	1.01 (0.9-1.03)	
Outpatient visits in past 12 months	
0	1.00	1.00
1–4	1.28 (0.84–1.96)	1.14 (0.80–1.63)
5 or more	1.94 (1.34–2.81)***	1.93 (1.48–2.51)***

***p < 0.001; **p < 0.01; *p < 0.5.

Overall, 30.3% of older hypertensive people were aware of their diagnosis, 24.8% of older hypertensives were taking treatment in the past two weeks to lower their blood pressure, and 48.8% of those who were taking antihypertensive treatment had their blood pressure controlled. Women, older age and more frequent out-patient visits in the past 12 months were associated with awareness of their hypertensive status and were taking treatment to lower their blood pressure in the past two weeks. However, there were no statistical differences in gender, age and out-patient visits among those who were taking antihypertensive treatment and had their blood pressure controlled. Of the total 2 841 hypertensive participants, 1 081 (38.1%) were aware of their hypertension, 985 (32.7%) were being treated, and 486 (17.1%) had their hypertension under controll (Table 3).

**Table 3:** Multivariate Logistic Regression Of Awareness, Treatment And Control Rates Of Hypertension Among Older Adults In South Africa

	*Awareness (30.3%)*	*Treatment (24.8%)*	*Control of treated (48.8%)*
	*AOR (95% CI)*	*AOR (95% CI)*	*AOR (95% CI)*
Gender			
Female	1.00	1.00	1.00
Male	0.68 (0.51–0.90)**	0.67 (0.50–0.88)**	1.09 (0.81–1.46)
Age			
50-59	1.00	1.00	1.00
60-69	1.70 (1.16–2.48)**	1.89 (1.38–2.58)***	0.73 (0.55–0.95)
70 and over	1.77 (1.22–2.59)**	2.15 (1.40–3.30)***	0.73 (0.50–1.06)
Population group			
African black	1.00	1.00	1.00
White	0.81 (0.40–1.62)	0.97 (0.52–1.83)	0.89 (0.44–1.80)
Coloured	1.00 (0.65–1.52)	1.09 (0.73–1.63)	0.80 (0.58–1.10)
Indian or Asian	1.08 (0.56–2.07)	1.45 (0.73–2.91)	1.25 (0.64–2.45)
Marital status			
Single	1.00	1.00	1.00
Married	1.38 (0.83–2.29)	1.50 (0.85–2.66)	1.44 (0.90–2.29)
Separated/divorced	1.17 (0.69–1.97)	1.11 (0.62–1.99)	1.11 (0.65–2.45)
Widow	1.67 (0.96–2.92)	1.45 (0.81–2.62)	1.51 (0.93–2.46)
Educational level			
No schooling	1.00	1.00	1.00
Less than primary	0.96 (0.65–1.52)	1.03 (0.67–1.58)	0.99 (0.64–1.54)
Primary	1.10 (0.72–1.68)	1.03 (0.65–1.61)	1.04 (0.73–1.49)
Secondary	0.94 (0.65–1.38)	1.09 (0.75–1.59)	1.54 (1.04–2.27)*
Wealth			
Low	1.00	1.00	1.00
Medium	1.43 (0.98–2.08)	1.36 (0.84–2.20)	0.75 (0.49–1.15)
High	1.55 (1.00–2.40)*	1.34 (0.75–2.40)	0.93 (0.64–1.33)
Geolocality			
Rural	1.00	1.00	1.00
Urban	1.27 (0.95–1.71)	1.30 (0.84–2.01)	1.17 (0.87–1.58)
Outpatient visits in past 12 months			
0	1.00	1.00	1.00
1–4	1.85 (1.10–3.11)*	1.94 (1.20–3.12)**	0.85 (0.61–1.17)
5 or more	4.49 (3.02–6.66)***	5.95 (3.96–8.95)***	0.87 (0.63–1.19)

***p < 0.001; **p < 0.01; *p < 0.5.

## Discussion

The study found significant rates of hypertension of 77.3% (male 74.4%, female 79.6%) among older adults (50 years and older) in South Africa. These rates seemed to be higher than in a previous survey in South Africa (men 44.0–52.0% and women 51.6–60.4%) in 1998,^3^ and similar to other studies such as in urban Senegal (65.4%),^4^ urban Zimbabwe (72%),^5^ and Turkey (71.2–82.2%).^12^ The rates were higher than in a number of other countries including rural Malawi, Rwanda and Tanzania (36.6–41.0%),^6^ Brazil (55%),^9^ China (24.2–64.9%),^10^,^11^ and Taiwan (31.1–38.0%).^13^

The study further found that regarding socio-demographics, with multivariate analysis, being in the Coloured population group was associated with higher rates of hypertension. This confirms previous studies in women.^3^ Further initiatives are required to address the high rate of hypertension in this population group. Unlike in other studies,^13^-^15^,^17^ this study did not find any effect of gender, age, level of education and geolocality on hypertensive status.

In terms of health variables, this study was in agreement with other studies,^4^,^10^,^13^,^14^,^18^,^31^ that having had a stroke, being overweight and having had more out-patients care visits in the past 12 months were associated with hypertension. Unlike some other studies,^10^,^13^,^21^,^23^-^25^,^33^ this study did not find insufficient fruit and vegetable intake, smoking, being physically inactive and having more limitations in activities of daily living to be associated with hypertension. The finding that current alcohol use was protective for hypertension in this study may be in line with some previous studies where light-to-moderate alcohol consumption decreased hypertension risk in women and increased the risk in men.^21^

The rates of awareness, treatment and control among the hypertensive participants in the present study (38.1, 32.7 and 17.1%, respectively) were as low as in some studies in rural China (35.2, 28.7 and 1.0%),^10^ Senegal (49.5, 37.0 and 5.7%),^4^ and Italy (65.6, 59.5 and 10.5%).^18^ They were however lower than in studies in Costa Rica (74.9% aware and more than half controlled)^7^ and in the USA (74% aware and 51.6% controlled among men and 37% controlled among women).^34^

As found in some other studies, in this study, women were more frequently aware of their hypertension and more frequently treated.^7^,^35^ They were not significantly more frequently controlled than men, though, as found in other studies.^7^,^36^ In the group of individuals who were unaware of their hypertension, it may have been because of multiple factors, such as never having been screened for hypertension, having been previously diagnosed but had forgotten the diagnosis, the medical provider had not considered their blood pressure levels to be sufficiently elevated to warrant the diagnosis, or had had inadequate health education and limited access to healthcare services.^9^,^35^ Therefore, more efforts, such as public health education and a blood pressure-monitoring system should be included for the older age group, particularly men, to improve their unsatisfactory awareness, treatment and control of hypertension.^10^

## Limitations of the study

This study had several limitations. Firstly, the self-report of health variables such as tobacco or alcohol use should be interpreted with caution; it is possible that respondents under-reported, especially females. As in many studies, arterial blood pressure was measured three times during a single session (two hours), which may have led to an overestimation of the prevalence of hypertension. In addition, the awareness and treatment rate of hypertension was solely assessed by individual self-report. Furthermore, this study was based on data collected in a crosssectional survey. We cannot, therefore, ascribe causality to any of the associated factors in the study.

## Conclusion

This study revealed high rates of hypertension among older adults (50 years and more) in South Africa, which put them at risk for cardiovascular disease. The percentages of hypertensives who were aware of, treated for and controlled were very low. These data underscore the urgent need to strengthen the public health education and blood pressure-monitoring systems to better manage hypertension among older adults in South Africa. Community healthcare workers in their new role in South Africa could screen for hypertension among older adults using a primary care ‘high-risk’ approach once every two years. This screening process would enable the health system to identify and cater for the needs of this vulnerable population group.^37^
